# Memory deficits of MDMA users are linked to cortical thinning related to 5-HT receptor densities

**DOI:** 10.1093/brain/awaf391

**Published:** 2025-10-22

**Authors:** Rebecca C Coray, Vincent Beliveau, Josua Zimmermann, Katrin H Preller, Michael Wunderli, Markus R Baumgartner, Erich Seifritz, Ann-Kathrin Stock, Christian Beste, David M Cole, Boris B Quednow

**Affiliations:** Experimental Pharmacopsychology and Psychological Addiction Research, Department of Adult Psychiatry and Psychotherapy, University Hospital of Psychiatry Zurich, University of Zurich, 8032 Zurich, Switzerland; Neuroscience Center Zurich, ETH Zurich and University of Zurich, 8057 Zurich, Switzerland; Neurobiology Research Unit, Copenhagen University Hospital Rigshospitalet, 2100 Copenhagen, Denmark; Department of Neurology, Medical University of Innsbruck, 6020 Innsbruck, Austria; Experimental Pharmacopsychology and Psychological Addiction Research, Department of Adult Psychiatry and Psychotherapy, University Hospital of Psychiatry Zurich, University of Zurich, 8032 Zurich, Switzerland; Neuroscience Center Zurich, ETH Zurich and University of Zurich, 8057 Zurich, Switzerland; Experimental Pharmacopsychology and Psychological Addiction Research, Department of Adult Psychiatry and Psychotherapy, University Hospital of Psychiatry Zurich, University of Zurich, 8032 Zurich, Switzerland; Experimental Pharmacopsychology and Psychological Addiction Research, Department of Adult Psychiatry and Psychotherapy, University Hospital of Psychiatry Zurich, University of Zurich, 8032 Zurich, Switzerland; Center for Forensic Hair Analytics, Institute of Forensic Medicine, University of Zurich, 8057 Zurich, Switzerland; Department of Adult Psychiatry and Psychotherapy, University Hospital of Psychiatry Zurich, University of Zurich, 8032 Zurich, Switzerland; Cognitive Neurophysiology, Department of Child and Adolescent Psychiatry, Faculty of Medicine, TU Dresden, 01307 Dresden, Germany; Cognitive Neurophysiology, Department of Child and Adolescent Psychiatry, Faculty of Medicine, TU Dresden, 01307 Dresden, Germany; Experimental Pharmacopsychology and Psychological Addiction Research, Department of Adult Psychiatry and Psychotherapy, University Hospital of Psychiatry Zurich, University of Zurich, 8032 Zurich, Switzerland; Translational Psychiatry, University Psychiatric Clinics Basel, University of Basel, 4056 Basel, Switzerland; Experimental Pharmacopsychology and Psychological Addiction Research, Department of Adult Psychiatry and Psychotherapy, University Hospital of Psychiatry Zurich, University of Zurich, 8032 Zurich, Switzerland; Neuroscience Center Zurich, ETH Zurich and University of Zurich, 8057 Zurich, Switzerland

**Keywords:** amphetamine-type stimulants, ecstasy, entactogens, empathogens, recollection, neurotoxicity

## Abstract

Regular recreational use of 3,4-methylenedioxymethamphetamine (MDMA, ‘ecstasy’) has been consistently linked to verbal memory dysfunctions, whose neurobiological origins remain unclear. Although neurochemical, structural and functional alterations resulting from repeated MDMA intake have been identified in user populations, only limited knowledge exists regarding potential interrelationships among these components and their implications for mnemonic functions.

The present study, thus, first examined the association of MDMA use with grey matter volume and cortical thickness. Subsequently, structural brain alterations were related to verbal memory performance and atlas-derived cerebral serotonin (5-HT) receptor expression patterns.

Our data, involving 122 participants (61 MDMA users, 61 age-, sex- and education-matched MDMA-naive controls), revealed a robust reduction of grey matter volume within hippocampal regions and impaired verbal learning, short-term recall after interference, long-term recall and recognition performance in MDMA users compared with controls. Self-reported MDMA use in the past 6 months correlated with several memory performance scores. A moderate inverse correlation across all participants was observed between hippocampal volumes and verbal long-term memory. Correspondingly, hippocampal CA1 grey matter volume was related to MDMA use intensity in the last months as indicated by MDMA hair concentrations. The extent of cortical and subcortical grey matter differences between groups correlated with atlas-based 5-HT_1A_, 5-HT_2A_ and 5-HT_4_ receptor densities, suggesting a serotonergic basis for mnemonic and structural alterations in MDMA users.

Together, these findings highlight a multidimensional origin of memory dysfunction in MDMA users, where alterations in the chemoarchitecture of the brain may affect behaviour, possibly via influences on brain grey matter structures.

## Teaser

Regular MDMA use is linked to memory dysfunction, but the underlying mechanisms are unclear. Coray *et al*. found reduced hippocampal grey matter volume in users, which correlated with memory deficits and serotonin receptor maps, suggesting that MDMA-related memory problems stem from both structural and neurochemical changes.

## Introduction

3,4-Methylenedioxymethamphetamine (MDMA) is among the most frequently used amphetamines for recreational purposes.^1,2^ It has gained popularity in clubs and other social settings because of its capacity to induce pleasant psychological states with heightened euphoria, sociability and sensory perception. However, negative neuropsychiatric side effects of chronic use, particularly long-term cognitive impairment and at least transient mood disturbances, are well-documented.^3,4^ Verbal declarative memory deficits, with moderate to large effect sizes, have been consistently shown, primarily in studies involving chronic and intense MDMA users.^5-9^

The acute behavioural effects of MDMA are mediated via the excessive release of serotonin (5-HT), noradrenaline, and to a lesser extent, dopamine.^10,11^ A temporary deficiency of 5-HT is held responsible for depressed moods, sleep disturbances and anxiety often occurring post-acutely (‘midweek blues’).^12^ Animal models across different species demonstrated that high doses of MDMA have long-term neurotoxic effects selectively affecting the 5-HT system, such as dendrite loss of cortical serotonergic neurons.^10,13^ Chronic MDMA use in humans has been associated with enduring hypofunction of the 5-HT system, as revealed by PET studies, which demonstrated reduced serotonin transporter (SERT) binding in cortical brain regions among frequent MDMA users.^14,15^ Meta-analytic studies confirmed lower SERT-binding in the frontal, parietal, temporal, insular, occipital, and anterior cingulate cortex, thalamus and hippocampus.^16^ PET studies of 5-HT_2A_ receptor distributions in MDMA users found both upregulation and downregulation with the duration of abstinence suggested as a mediating factor. Accordingly, former MDMA users showed higher 5-HT_2A_ receptor densities in the occipital cortex, whereas recent MDMA users exhibited reduced densities across all examined cortical regions.^17^ Notably, while SERT availability has been shown to correlate with MDMA use intensity and seems to recover with sustained abstinence, memory function may remain impaired despite the restoration of serotonergic substrates.^18^ Given the comprehensive evidence of persistent cognitive impairments in chronic MDMA users, the risk of MDMA causing permanent neurotoxic damage to 5-HT axon terminals in humans cannot yet be ruled out.^10,13,15^ While numerous studies have identified deficits in executive function and memory, along with alterations in the 5-HT system following chronic MDMA use,^7^ direct correlations between these cognitive changes and serotonergic markers have not been consistently established. This inconsistency might be attributed to the impact of indirect factors, such as 5-HT-modulated neuroplasticity.^19,20^ 5-HT influences neuroplasticity through various molecular mechanisms, including the activation of neurotrophic proteins, such as the brain-derived neurotrophic factor,^21^ and intracellular signalling cascades that induce cytoskeletal rearrangement in neuronal cells.^22^ Additionally, 5-HT governs structural plasticity via glutamatergic transmission and *N*-methyl-D-aspartate (NMDA) receptor-dependent long-term potentiation.^23^ Changes in neuroplasticity in humans can be indicated by alterations in grey matter volume, as measured by structural MRI *in vivo*.^24^ For example, increases in grey matter volume in certain brain regions have been observed following chronic intake of 5-HT reuptake inhibitors as part of psychiatric treatments.^22^ Moreover, a positive association has been reported between 5-HT_1A_ receptor binding and grey matter volume in the hippocampus and the temporal cortices in healthy participants.^25^ However, only two studies to date have examined grey matter volume alterations in recurrent MDMA users,^26,27^ while no study has investigated the association between grey matter changes and memory performance or the distribution density of receptors and transporters of the 5-HT system in this population.

Here, we aimed to investigate whether chronic MDMA users display structural brain alterations, as indicated by cortical thickness and volume, in specific brain regions compared with age-, sex-, verbal intelligence quotient (IQ)- and education-matched MDMA controls. We correlated cortical and subcortical grey matter volume differences with atlas-derived distribution densities of 5-HT receptors and transporters in the healthy human brain.^28^ We hypothesized that grey matter volume decreases in MDMA users would: (i) be observed specifically in brain regions that are known to subserve memory functions, such as the prefrontal cortex and the medial temporal lobe; and (ii) occur within regions known for high expression of specific 5-HT receptors and SERT. Moreover, (iii) we expected participant verbal memory performance to be associated with cortical thinning in brain regions richly expressing 5-HT receptors and SERT. Finally, (iv) we anticipated grey matter abnormalities and memory impairment to be associated with MDMA use intensity, indicating a potential pathological consequence of use.

## Materials and methods

### Study design

This study employed a cross-sectional, matched case-control design. The data presented in this article stem from two previous studies, both conducted at the University Hospital of Psychiatry Zurich. The data collection took place in 2015–16,^9^ and 2019–21,^6,29^ respectively. Both studies routinely obtained T1-weighted structural images using the same scanner and with the same sequence and have not yet been separately analysed and published. We combined the structural image data of both studies, along with all additional assessments from both that were gathered with identical methodologies. With this, we gained a comprehensive dataset including 132 participants (64 MDMA users and 68 controls), structural imaging data, a verbal memory test, psychometric characterization and substance use measurements including hair toxicology and self-reports. Both studies were approved by the Cantonal Ethics Committee of Zurich, Department of Health of the Canton of Zurich, Switzerland (E-14/2009, Amendment 2 and BASEC 2018-02125).

### Participants

Both studies followed a similar recruitment procedure. Eligible individuals were invited to undergo an assessment at the University Hospital of Psychiatry Zurich. MDMA-naive controls were matched to MDMA users, ensuring congruence in terms of sex, age, verbal IQ and education across both studies. Notably, both investigations exclusively enrolled regular MDMA users who showed limited co-use of other substances. However, there were minor differences in the inclusion criteria between the two studies, which are described in the Supplementary material. In both studies, toxicological hair tests were conducted to confirm self-reported MDMA and other substance use within recent months (see later). Both investigations excluded individuals with a history of heavy cannabis consumption and those who reported having used heroin intravenously once in their lifetime. Participants with higher cocaine or amphetamine than MDMA concentrations in the hair were excluded. Furthermore, for controls, individuals who had consumed illicit substances on more than 15 occasions in life (except cannabis) were also ineligible for participation in both studies. Additional exclusion criteria encompassed participants diagnosed with psychiatric axis I disorders or with a family history of schizophrenia, bipolar disorder or obsessive-compulsive disorder according to Diagnostic and Statistical Manual of Mental Disorders IV (DSM-IV). Participants who indicated clinically significant somatic illness, neurologic disorder or head injury were also excluded, as well as individuals reporting the usage of prescription medications affecting the central nervous system. Lastly, current pregnancy constituted another criterion for exclusion.

### Clinical and substance use assessments

To exclude psychiatric axis I disorders, the Structured Clinical Interview of the DSM-IV (SCID-I)^30^ and Beck Depression Inventory^31^ were used for the investigation in 2015–16. For the investigation in 2019–21, the Mini-International Neuropsychiatric Interview^32^ and the Center for Epidemiologic Studies Depression Scale^33^ were used. Both studies used the Attention-Deficit/Hyperactivity Disorder Self-Rating Scale^34^ to assess attention deficit hyperactivity disorder (ADHD) symptoms and a German Vocabulary Test (MWT-B)^35^ to estimate verbal IQ. Substance use was estimated with the standardized and structured Interview for Psychotropic Drug Consumption,^36^ which assesses the frequency, quantity and duration of present and past use of a broad range of psychotropic substances.

#### Rey Auditory Verbal Learning Test

We applied the German equivalent^37^ of the Rey Auditory Verbal Learning Test (RAVLT)^38^ to assess verbal declarative memory. It consists of ‘list A’ of 15 nouns that participants are asked to memorize after each of five encoding trials. The subsequent assessment necessitates retrieval at two distinct time points: after a brief interference ‘list B’ and following a 2-h delay recall. After the delayed recall participants had to recognize the nouns of list A from a list of 50 distractor words. Supraspan (first encoding trial), learning performance (sum of totally learned words after five trials), recall of list A after interference list B (recall after presentation of the interference list), delayed recall (recall of list A after 2 h) and recognition (probability of correctly discriminating words from list A among lures) were extracted as dependent variables.^39^

#### Toxicological hair and urine testing

Toxicological hair analyses were conducted by employing liquid chromatography-tandem mass spectrometry.^40^ Within both studies, the hair concentrations of MDMA, cocaine, amphetamine and a range of other substances were quantitatively assessed in hair samples of 3–4 cm length, covering a retrospective period of 3–4 months. Urine samples were analysed with immunoassay-based methods.^6^

### Study procedures

The protocols employed in both studies only slightly diverged in terms of tasks and interviews tailored to their respective research inquiries. Initially, the participants provided written informed consent. Thereafter, the examiner conducted a demographic questionnaire and the Interview for Psychotropic Drug Consumption. This was followed by study-specific clinical and cognitive assessments, before undergoing MRI investigation. After completing all tasks, participants were compensated with a monetary reward.

#### Image acquisition

Both studies used the same 3 T Philips Intera Achieva scanner equipped with a 32-channel head coil (Philips Medical Systems) located at the University Hospital of Psychiatry Zurich. Anatomical images were acquired using the same T1-weighted magnetization prepared rapid acquisition gradient (MPRAGE) sequence (field of view: 240 × 240 mm; number of slices: 160; resolution: 1 × 1 × 1 mm; repetition time: 2983 ms; echo time: 3.7 ms; inter echo delay: 8.1 ms; inversion time: 1004 ms; flip angle: 8°; bandwidth: 192 Hz/pixel).

#### MRI preprocessing

Anatomical images were processed with the CAT12 toolbox (version 12.8.2) within SPM12 (version 7771) using MATLAB (version R2020b). Images were preprocessed by using the standard preprocessing pipeline that is implemented in the CAT12 toolbox.^41^ The images were segmented and spatially normalized into standard Montreal Neurological Institute (MNI) space. In the output options, cortical surface, and cortical thickness estimation for region of interest (ROI) analysis were selected. According to the toolbox recommendations, the segmented grey matter images were smoothed with a 6 mm full-width at half-maximum Gaussian kernel. To correct for different brain sizes in statistical models, the total intracranial volume (TIV) was estimated. The mean cortical thickness values were extracted for 70 cortical ROIs defined by the Desikan–Killiany atlas (DK atlas).^42^ For subcortical regions, the mean cortical volumes were extracted by voxel-based morphometry (VBM)^24^ for 52 ROIs as defined by the Computational Brain Anatomy (COBRA) atlas.^43^ For ROI extraction, the standard procedures of the CAT12 toolbox were used.^41^ The estimated mean cortical thickness and cortical volume values for each ROI were then averaged between hemispheres as no lateralization effects were expected. The cerebellum and subcortical ROIs defining white matter regions were not considered as ROIs and therefore omitted from the analysis. This resulted in a total of 34 cortical ROIs, along with an additional eight subcortical ROIs.

### Statistical analysis

Analyses were carried out with R (version 4.1.2).^44^ In all regression and analysis of covariance (ANCOVA) models described later, *P*-values were Benjamini–Hochberg corrected for the number of respective models. Additional statistical information is available in the Supplementary material.

#### Group differences in structural imaging

Linear regression models were employed to investigate group differences in grey matter thickness and volumes across the 42 brain regions with the respective brain region as the dependent variable and group as the independent variable of interest, while age, sex and weekly amounts of alcohol (g/week), cannabis (mg/week) and nicotine (cigarettes/week) use were introduced as covariates. For the subcortical regions, TIV was included as an additional covariate.

#### Group differences in memory performance

For the scores of learning performance, recall after interference, recall after 2 h and recognition, the assumption of normality was not met. Therefore, five permutation covariance models (ANCOVA) with group as an independent variable and the respective memory index as a dependent variable were calculated using the lmPerm package (version 2.1.0).^45^ Additionally, age, sex, verbal IQ and the weekly use of alcohol, cannabis and nicotine were included as control variables.

#### Correlation of structural imaging and memory

The associations between brain regions and memory scores both with significant group differences was analysed with ANCOVA models. Memory indices were dependent variables. As an independent variable, the interaction Group × Brain region was included, with the control variables age, sex, verbal IQ and weekly use of alcohol, cannabis and nicotine. In the case of non-significance, the interaction term was dropped from the analysis, and results without the interaction term are reported.

#### Correlations with PET-derived 5-HT receptor and transporter maps

To investigate associations between cortical and subcortical grey matter differences and 5-HT transporter and receptor distributions, the differences in cortical thickness and subcortical volumes between groups (controls minus MDMA users) were correlated with the regional density of published PET maps for SERT, 5-HT_1A_, 5-HT_1B_, 5-HT_2A_ and 5-HT_4_^28^ (each separately).

#### Correlations with substance use intensity

The association of past substance use with structural brain alterations showing group differences (dependent variables) was analysed with regression models only within MDMA users. Control variables included age, sex and verbal IQ. Weekly amounts (milligrams or grams), as well as hair concentrations of each substance (MDMA, alcohol, nicotine, cannabis, amphetamine, cocaine), were treated as independent variables, each in a separate model (12 models in total), to mitigate intercorrelation issues. The relation of past substance use with memory was investigated using the same approach.

#### Sensitivity power analysis

For multiple regression analyses with up to 10 predictors, an assumed power of 80%, and an alpha-error probability of 5%, we can detect small effects with *f*^2^ > 0.065 in our sample of *N* = 122. In ANCOVA models (two groups, up to 10 covariates) with similar assumptions, we are able to detect moderate effects sizes of *f*^2^ > 0.256. In correlation analyses with the full sample we can detect weak associations with *r* > ±0.178, while in MDMA users only (*N* = 61) weak correlations with *r* > ±0.252 are detectable.

## Results

Data of three MDMA users and seven controls were excluded due to incomplete demographic information or incomplete substance use data. Accordingly, the final analysis included a total of 122 participants (61 MDMA users and 61 controls).

### Demographic structure and substance use

MDMA users and controls did not differ regarding sex distribution, age, verbal IQ, years of school education, number of smokers and ADHD and depression symptoms (Table 1). However, MDMA users smoked more and for longer and reported higher and longer alcohol consumption compared with controls. In addition, MDMA users reported more intense use of cannabis, cocaine and amphetamines than controls; however, the intake of these substances was lower and less regular compared with MDMA. Two control participants consumed MDMA once in their lifetime; however, this occurred more than 6 years ago, and the reported amount was negligible (<0.1 g). Of note, 6 of the 122 participants were left-handed (two MDMA users, four controls).

**Table 1:** Demographics and substance use characteristics

	MDMA users	Controls	Statistic	*P*
Sex, *n*				
Male	26	29	*χ* ^2^ = 0.132	0.716
Female	35	32		
Age, years	28 (9)	27 (10)	*W* = 1724	0.484
Years of school education	9 (3)	10 (3)	*W* = 1728	0.452
Verbal IQ	29 (68)	30 (70)	*W* = 2099	0.221
ADHD-SR symptom score	10 (11)	9 (10)	*W* = 1642	0.262
Depression score	0 (1)	0 (1)	*W* = 923	0.997
MDMA, days since last consumption	25 (154)	2775 (355)		
MDMA use, mg/week	74.4 (99)	0 (0)		
MDMA use, years	8 (9)	0 (0)		
MDMA lifetime gram	29.74 (46)	0 (0)		
MDMA hair concentration, pg/mg	819 (3055)	0 (2)		
Alcohol use, g/week	119 (162)	52 (102)	*W* = 1095	<0.001***
Alcohol use, years	10 (13)	7 (13)	*W* = 1406	0.020*
Number of smokers, *n*	37	28	*χ* ^2^ = 2.667	0.102
Nicotine occasions	59 (526)	0 (140)	*W* = 1473	0.038*
Nicotine use, years	7 (14)	2 (10)	*W* = 1345	0.007**
Cannabis use, mg/week	11.5 (142)	0 (2)	*W* = 1060	<0.001***
Cannabis use, years	6 (11)	0 (5)	*W* = 1059	<0.001***
Amphetamine use, mg/week	1.0 (26)	0 (0)	*W* = 947	<0.001***
Amphetamine use, years	0 (5)	0 (0)	*W* = 1007	<0.001***
Amphetamine hair concentration, pg/mg	0 (140)	0 (1)	*W* = 947	<0.001***
Cocaine use, mg/week	1.9 (30)	0 (0)	*W* = 907	<0.001***
Cocaine use, years	0 (7)	0 (0)	*W* = 976	<0.001***
Cocaine hair concentration, pg/mg	137 (715)	5 (26)	*W* = 758	<0.001***

Values are presented as *n* or median (interquartile range, IQR) (interquartile range: third quartile—first quartile). *P*-values for group comparisons are reported, as the data were non-normally distributed. mg/week = milligrams for the previous 6 months. Years = years of use. Occasions = weekly occasions for the previous 6 months. ADHD-SR = Attention-Deficit/Hyperactivity Disorder Self-Rating Scale; IQ = intelligence quotient; Lifetime gram = estimated lifetime consumption in grams; MDMA = 3,4-methylenedioxymethamphetamine. *P < 0.05; **P < 0.01; ***P < 0.001.

### Group differences in structural imaging

**Figure 1:**
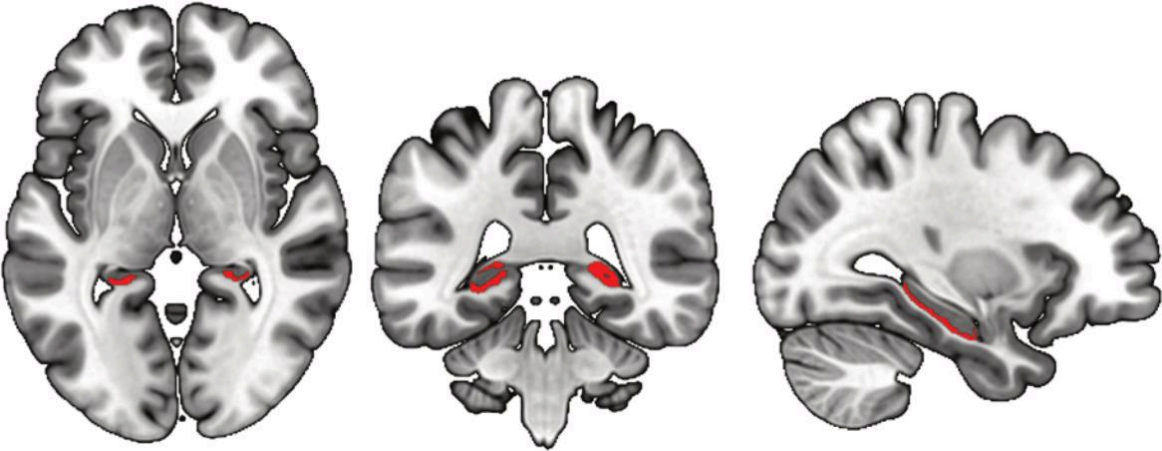
**Group differences in structural imaging.** Significant differences in hippocampal brain regions after applying Benjamini–Hochberg correction for the comparison of 42 different regions of interest between 3,4-methylenedioxymethamphetamine (MDMA) users and controls.

The group differences in the hippocampal regions CA1 and CA2/CA3 regions remained significant after correction for 42 comparisons (Fig. 1): MDMA users showed reduced grey matter volume in CA1 than controls [*β* = −0.036, *t*(114) = 3.94, *P* < 0.001, *P*_corrected_ = 0.006], with a medium to large effect size (*f*^2^ = 0.14). Also, grey matter volume of the hippocampal CA2/3 area was significantly lower in MDMA users [*β* = −0.006, *t*(114) = 3.16, *P* = 0.002, *P*_corrected_ = 0.042, *f*^2^ = 0.09]. Significance at the uncorrected level was observed for the hippocampal CA4 region, the lateral occipital cortex and the isthmus of the cingulate gyrus (see Supplementary Tables 1 and 2 for further details).

### Group differences in memory

**Figure 2:**
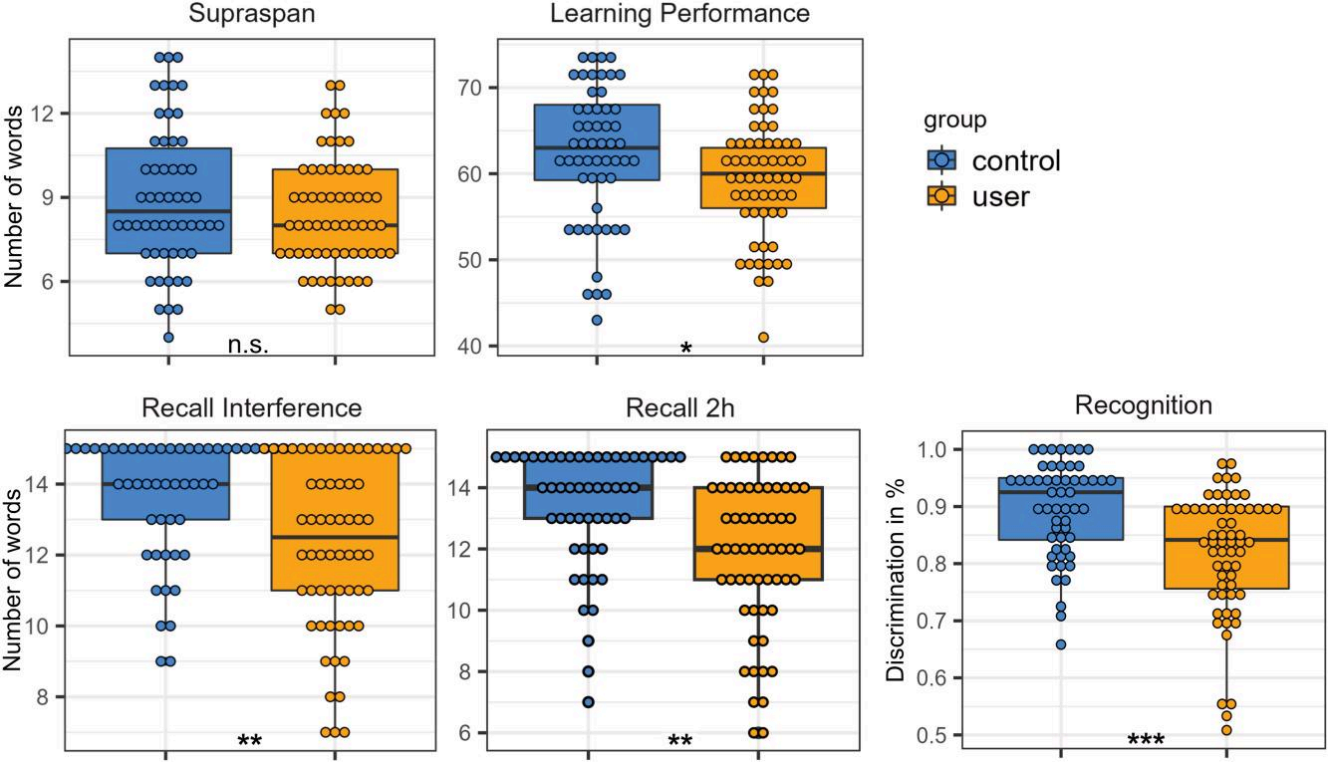
**Group differences in memory.** Memory performance scores in 3,4-methylenedioxymethamphetamine (MDMA) users (orange) and MDMA-naive controls (blue). Results are Benjamini–Hochberg corrected. n.s. = not significant; *P < 0.05; **P < 0.01; ***P < 0.001.

Some of the memory data was previously published separately for both samples,^6,9^ but not yet as pooled data (Fig. 2 and Supplementary Table 3). Group differences were found for learning performance [*F*(1,288) = 6.25, *P =* 0.014, *P*_corrected_ = 0.040, *f*^2^ = 0.07], recall after interference [*F*(1,65) = 16.45, *P* < 0.001, *P*_corrected_ = 0.001, *f*^2^ = 0.16], recall after 2 h [*F*(1,58) = 12.54, *P* < 0.001, *P*_corrected_ = 0.002, *f*^2^ = 0.12], as well as recognition [*F*(1,0.17) = 21.83, *P* < 0.001, *P*_corrected_ < 0.001, *f*^2^ = 0.21]. In contrast, the supraspan did not show a significant group difference [*F*(1,10) = 2.15, *P =* 0.145].

### Association between hippocampal volume and memory

Next, we examined the association between memory performance and hippocampal CA1 and CA2/3 volumes and whether this association differed between groups. Hippocampal volumes did not reveal a significant prediction for any memory score with the factor group in the model. However, the group variable remained significant for each model, except with supraspan (Supplementary Tables 4 and 5). Therefore, we calculated the same models for the entire sample, omitting the group factor. Then, hippocampal CA2/3 volume was significantly associated with recall after 2 h after correcting for five comparisons [*F*(1,35) = 7.24, *P* = 0.008, *P*_corrected_ = 0.041, *f*^2^ = 0.07]. Further associations showed significance at the uncorrected level but were marginally not significant at the corrected level: recall after 2 h was also positively correlated with hippocampal CA1 volume [*F*(1,27) = 5.51, *P* = 0.021, *P*_corrected_ = 0.051, *f*^2^ = 0.05], while recall after interference was positively associated with hippocampal CA1 volume [*F*(1,12) = 5.53, *P* = 0.021, *P*_corrected_ = 0.051, *f*^2^ = 0.05] and CA2/3 volume [*F*(1,22) = 5.07, *P* = 0.026, *P*_corrected_ = 0.066, *f*^2^ = 0.06].

### Correlation of structural imaging differences with 5-HT receptor and transporter distributions

**Figure 3:**
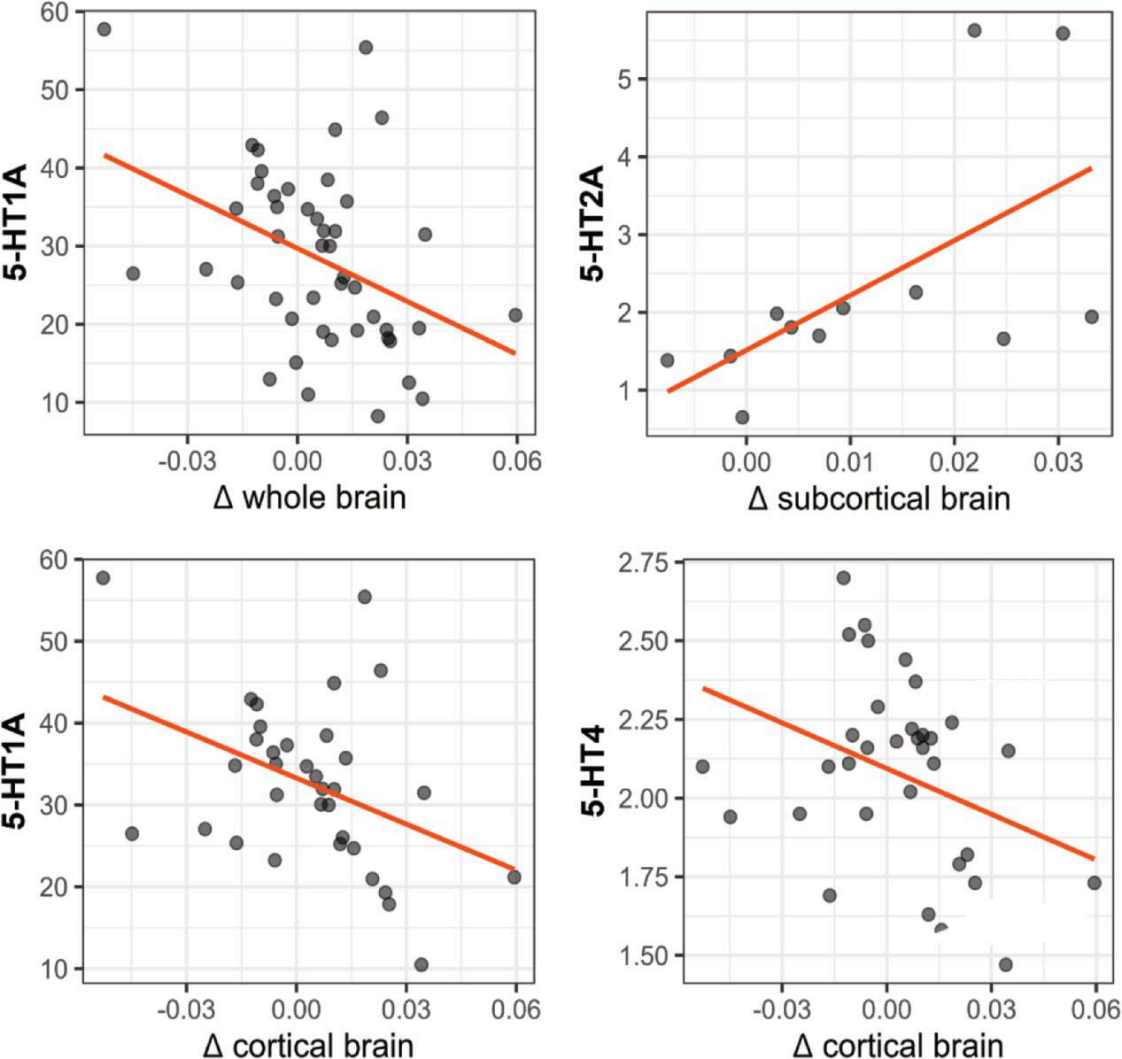
**Correlation of structural group differences with serotonergic markers.** Pearson correlations with differences grey matter thickness/volume and 5-HT receptor densities.

Only the 5-HT_1A_ receptor was unimodally distributed across both cortical and subcortical regions. Accordingly, the correlation of 5-HT_1A_ receptor densities with the combined cortical and subcortical cortical thickness and volume values was statistically significant even after applying Benjamini–Hochberg corrections for five comparisons [*r* = −0.398, *P =* 0.006, *P*_corrected_ = 0.031, 95% CI (−0.616, −0.212)]. Within cortical regions, the difference in cortical thickness between MDMA users and controls was significantly correlated with 5-HT_1A_ receptor [*r =* −0.404*, P =* 0.017, 95% CI (−0.653, −0.076)] and 5-HT_4_ receptor distributions [*r =* −0.350*, P =* 0.042, 95% CI (−0.615, −0.013)], only at uncorrected levels. Within subcortical regions, only the 5-HT_2A_ receptor distribution was significantly correlated with volume over all regions at the uncorrected level [*ρ =* 0.643*, P =* 0.028, 95% CI (0.433, 0.787)] (see Fig. 3 and Supplementary Table 6 for details). SERT distributions were not correlated with structural alterations of MDMA users. Supplementary Table 7 presents spatial autocorrelation-corrected results, which are consistent with those reported above.

### Associations between hippocampal volume and substance use

#### MDMA hair concentration

We found a small, but significant inverse association between MDMA hair concentration and CA1 volume [*β* = −0.006, *t*(0.003) = 2.17, *P* = 0.034, *f*^2^ = 0.09]. The association between MDMA hair concentration and CA2/3 volume didn’t yield significance. In both models, the effect of sex was significant, with females having smaller hippocampal volume (CA1: *β* = −0.054, *P* < 0.001, *f*^2^ = 0.51; CA2/3: *β* = −0.008, *P* = 0.004, *f*^2^ = 0.16). There was no interaction between brain regions and sex. Removing sex from the models increased the effect size of the association between MDMA hair concentration and hippocampal volumes (CA1: *β* = − 0.011, *P* = 0.002, *f*^2^ = 0.18; CA2/3: *β* = −0.002, *P* = 0.018, *f*^2^ = 0.10).

#### Self-reported substance use

Neither self-reported weekly use of MDMA nor of other substances (alcohol, nicotine, cocaine and amphetamine) revealed a significant association with hippocampal volumes in the MDMA user group. Again, a significant influence was revealed by sex, with females having smaller hippocampal volumes (CA1: *β* = −0.031, *P* < 0.001; CA2/3: *β* = −0.009, *P* = 0.001). There was no interaction between sex and self-reported MDMA consumption.

### Associations between memory and substance use

For previously significant memory indices, we analysed the association between memory performance and MDMA use. The models revealed a significant association between weekly MDMA use and learning performance [*β* = −16.85, *t*(50) = 2.32, *P* = 0.024], recall after interference [*β* = −5.067, *t*(50) = 2.04, *P* = 0.044, *f*^2^ = 0.09] and recall after 2 h [*β* = −5.734, *t*(50) = 2.19, *P* < 0.033, *f*^2^ = 0.09], yet not with recognition. No other substance was significantly associated with memory. No significant effects were observed for MDMA hair concentration or any other substances (*β* > 0.20, *P* > 0.05) on memory scores.

## Discussion

Our study examined MRI-derived grey matter volumes and their relation to 5-HT receptor density maps and memory performance in a large sample of MDMA users and demographically matched MDMA-naive controls. Structural imaging revealed that MDMA users exhibited significantly smaller hippocampal volumes within CA1 and CA2/3 regions of medium to large effect size. These findings survived alpha-error correction and the consideration of several confounders, such as alcohol, nicotine and cannabis co-use. In addition, the hippocampal volume of the CA4 region as well as the cortical thickness of the lateral occipital cortex and isthmus of the cingulate gyrus were different between groups but only at the uncorrected level. In accordance with our first hypothesis, we thus identified smaller grey matter volumes within the medial temporal lobe in MDMA users, a region relevant for verbal memory functions.^46,47^ A direct relationship between intensity of MDMA use and hippocampal volume atrophy was established by an inverse correlation found between hippocampal CA1 volume and the amount of MDMA consumed over the past 3 months, as assessed through hair analysis.

Consistent with our second hypothesis, the overall differences in total cortical and subcortical grey matter volume between MDMA users and controls was aligned with density maps of serotonergic markers. Specifically, we observed a strong relationship between grey matter differences among users and controls and the distribution of 5-HT_1A_ receptors across all brain regions. At uncorrected levels, cortical 5-HT_4_ receptor expression patterns, as well as subcortical 5-HT_2A_ receptor patterns, were also associated with cortical and subcortical grey matter differences, respectively.

Moreover, we again demonstrated memory impairment in these MDMA users at medium to large effect sizes^6,9^ and found that the amount of MDMA consumed in the last 6 months predicted the extent of memory dysfunction. This was true for learning performance, recall after interference, long-term recall and recognition. Importantly, no other substance (alcohol, nicotine, cannabis, amphetamine or cocaine) predicted memory performance in a similar way. While the cross-sectional nature of our study precludes causal inference, our findings align with prospective studies demonstrating that MDMA use can lead to changes in memory and hippocampal function over time. Accordingly, even low cumulative MDMA use was associated with declines in verbal memory performance in previously drug-naive individuals^48^ and impaired paired-associate learning within 12 months,^49^ a cognitive process strongly dependent on hippocampal integrity.^50^ Providing strong support for a causal link, a prospective functional MRI study demonstrated opposing trajectories of encoding-related activity in the left parahippocampal gyrus: it decreased over time in ongoing MDMA users but increased in abstinent individuals.^51^

Together, these findings suggest that grey matter alterations in MDMA users might be mediated by the 5-HT receptor system, which aligns with a number of studies in non-human primates,^52-54^ showing long-term neurochemical and structural changes in the serotonergic system after repeated MDMA administration, including reduced serotonin levels, destruction of serotonergic nerve terminals and abnormal reorganization of serotonergic axon projections. The link between MDMA-induced serotonergic alterations and loss of grey matter volume is reinforced by several animal studies across different species, in which MDMA-related serotonergic neurotoxicity was particularly evident and often persistent specifically in the hippocampus; however, neurotoxicity was also observed in cortical regions and the striatum.^55^ Of note, iron load as an indicator of possible neurotoxic processes was previously found to be increased in striatal regions of a subsample of the MDMA users investigated here.^56^

The CA1 region is densely innervated by serotonergic projections of the dorsal and ventral raphe nuclei.^57^ Acute MDMA intoxication was shown to exert cortical cell apoptosis via 5-HT_2A_ receptor stimulation in cerebral cortex cultures.^58^  *In vivo*, a similar effect was observed in the rodent hippocampus, where MDMA-induced activation of 5-HT_2A_ receptors increased glutamate efflux, resulting in the loss of interneurons.^59,60^ Collectively, these findings indicate a hyperexcitation of hippocampal neurons during the acute MDMA intoxication phase, particularly within a specifically susceptible hippocampal region, that might cause cell apoptosis.^61,62^ In line with this, 5-HT_2A_ antagonists have been found to attenuate MDMA-induced neurotoxicity.^63^ The translation of these findings from animal studies to humans, for instance, could be substantiated by the prior observation that recurrent MDMA users exhibit heightened cortical excitability in regions such as the occipital cortex, which was correlated with their lifetime consumption^64,65^ and increased 5-HT_2A_ expression.^17,66^ Interestingly, a similar observation was made in patients with Parkinson’s disease: greater grey matter volume loss was related to greater regional 5-HT_2A_ receptor availability.^67^ Such an observation aligns with our results, indicating a smaller grey matter volume in the MDMA-user group compared with a well-matched control group, where the extent of volume loss is related to 5-HT_2A_ subcortical receptor patterns. In contrast, our investigation yielded no differences in grey matter density between groups in prefrontal regions, even when considering the uncorrected results. Accordingly, our results are not completely in line with a previous study reporting grey matter reductions in the medial frontal and orbitofrontal cortex in repeated MDMA users.^26^ However, this inconsistency might be explained by either higher levels of MDMA use or stronger co-use of stimulants among assessed MDMA users in this previous study, with the latter being known to be associated with structural alterations, particularly in the prefrontal cortex.^27,68,69^ For this reason, we aimed to exclude participants with intense stimulant co-use in the present study. Our findings are not in direct conflict with prior studies reporting executive involvement in memory impairments among MDMA users, with some of these studies also employing the RAVLT,^5,39^ which assesses executive components of memory during learning and recall after interference.^38^ One study identified a frontal hypometabolism of glucose via fluorodeoxyglucose (FDG) PET in MDMA users, which was associated with learning and latter memory performance in the RAVLT, indicating a frontal contribution to the observed cognitive deficits.^5^ Interestingly, animal studies have demonstrated that activation of 5-HT_1A_ receptors increases frontal glucose metabolism, as measured by FDG-PET.^70^ This highlights the need for further investigations in humans, particularly to explore whether reduced 5-HT_1A_ receptor availability in MDMA users is associated with decreased cortical glucose metabolism. The influence of 5-HT_1A_ receptors on neuroplasticity has been highlighted in previous studies. In animal models, the role of 5-HT_1A_ receptors was delineated in mediating neurotrophic effects, second-messenger production and gene expressions involved in long-term potentiation and neuroplastic changes.^71^ Therefore, 5-HT_1A_ receptors were proposed to drive synaptic plasticity necessary for certain experience-driven brain changes, such as memory or learning.^72^ In humans, previous evidence demonstrated a strong association between 5-HT_1A_ receptor density and grey matter volume, particularly in the hippocampus and temporal, orbitofrontal and parietal cortical regions.^25,73^ In line with this, the present investigation uncovered an inverse association between the grey matter differences of controls and MDMA users and 5-HT_1A_ receptor expression patterns derived from PET maps of 5-HT receptors. Moreover, recent meta-analytic findings report an association between lower regional 5-HT_1A_ receptor availability and lower grey matter volume in Parkinson’s disease.^67^ In accordance, studies that investigated structural and neurochemical changes in patients with major depression described cortical and hippocampal grey matter volume loss,^74,75^ while antidepressant treatment, promoted via 5-HT_1A_-mediated neurotrophic/growth factors,^76^ led to the reversal of grey matter deficits.^77^

For the 5-HT_4_ receptor, there is extensive evidence highlighting its function in synaptic plasticity, memory and cognition.^20,78^ It is a potential target for treating cognitive impairment as shown in animal studies, where 5-HT_4_ receptor agonists enhanced hippocampal-dependent memory processes. Additionally, in humans, acute administration of the 5-HT_4_ receptor agonist prucalopride in young healthy participants improved recall and increased neural activation in the hippocampus and functionally related areas, relative to placebo.^79,80^ No previous study has related 5-HT_4_ expression densities to grey matter volume, although in animal models improved learning after 5-HT_4_ activation was found to be accompanied by dendritic spine growth and increased experience-dependent structural plasticity in learning-activated hippocampal circuits.^81^ While several PET studies have shown alterations in SERT occupancy in MDMA users,^16^ we did not find an association of grey matter changes and PET-derived SERT distributions in the current user cohort. However, it has been demonstrated that SERT expression can recover in MDMA users while memory deficits remain.^18^ Moreover, the memory performance of MDMA users was not found to be associated with SERT binding measured using single-photon emission computed tomography.^82^ Thus, SERT changes might not be substantially involved in both grey matter alterations and memory problems in MDMA users.

### Limitations

Some limitations should be considered when interpreting the present results. First, our recruitment procedure ensured that stimulant co-use was kept at the lowest possible level, but it was nevertheless present. The demographic comparisons revealed no significant differences in sex distribution, age, years of education, number of smokers, ADHD scores, verbal IQ or depression scores between the two groups. Nevertheless, MDMA users exhibited higher amounts of smoking, longer smoking duration, higher weekly alcohol consumption and more lifetime use of alcohol, cannabis, cocaine and amphetamine. We did not include cocaine and amphetamine use intensity as control variables in our models examining grey matter group differences, because both variables contained a lot of zero consumption values, particularly for the control group, potentially creating an artificial grouping variable without contributing statistically meaningful improvements to the results. However, we controlled for the potential influence of stimulants in a separate model within the MDMA group, confirming that the consumption of cocaine and amphetamine was not related to grey matter volume changes. Although we carefully controlled for the co-use of alcohol, cannabis and nicotine in our statistical models, and given that co-use of cocaine and amphetamine did not correlate with memory performance or structural brain changes, it remains possible that substance co-use contributed to the overall group effects on memory and grey matter volume.^83^ Accordingly, we cannot fully rule out that co-use of alcohol, nicotine, cannabis, cocaine, amphetamine and other substances impacted our results, although we controlled for most of those factors. Second, we conducted a purely cross-sectional analysis and thus, we could also not control for pre-existing structural grey matter differences; a longitudinal design would be needed to address this in future research. Finally, individual PET measurements were not available in our study and normative PET receptor density maps were instead used as a proxy. While the results derived from these data may capture underlying biological processes, it is important to underscore that, following the well-known ecological fallacy principle, these associations do not necessarily extend to the individual level. Still, recent studies using population-level receptor maps have yielded valuable, albeit indirect, insights into individual brain function.^84,85^

## Conclusion

Our study reveals significant disparities in the structure of the hippocampus (CA1 und CA2/3) between substantial and well-matched samples of MDMA users and MDMA-naive controls. Past MDMA consumption was related to both reduced memory performance and the amount of hippocampal grey matter thinning particularly in CA1, hinting at an association between verbal memory deficits and MDMA-induced 5-HT systemic alterations. Additionally, reductions in grey matter throughout the cortex and subcortex were found to be correlated with PET-derived density maps of the 5-HT_1A_, 5-HT_2A_ and 5-HT_4_ receptors. Thus, these effects might be attributed to mechanisms such as MDMA-induced serotonergic plasticity or, possibly, neurotoxicity, as indicated by reductions in grey matter macrostructure. This aligns with the observation that altered brain structure and function in psychiatric and neurological diseases correspond with alterations in chemoarchitecture.^86^ Consequently, our results are in line with the idea that modifications of the 5-HT system occurring in MDMA users can impact cognitive processes. The reduced grey matter volume found in the memory-relevant hippocampal regions of MDMA users was directly related to memory performance in our sample. However, a potential multifactorial origin of memory deficits aligns well with existing literature, which consistently identifies verbal memory impairments in MDMA users but describes several underlying neural origins, such as changes in frontal regional cerebral glucose metabolism, connectivity changes within multimodal sensory integration regions or global serotonin deficiency measured in CSF.^5,6,87,88^

## Supplementary Material

awaf391_Supplementary_Data

## Data Availability

The data that support the findings of this study are available from the corresponding author upon reasonable request. Any rights to data have been clarified among the authors.
