# Supplementary material for: Memory deficits of MDMA users are linked to cortical thinning related to 5-HT receptor densities
Source: Brain. 2025 Oct 22;149(4):1410–21. doi: 10.1093/brain/awaf391 (PMC13058468; doi:10.1093/brain/awaf391)
Supplement: awaf391_Supplementary_Data [file awaf391_supplementary_data.pdf]

Supplementary Material

Methods

Participants

The study performed in 2015-2016 required MDMA users to have consumed MDMA at least once within the previous six months and, additionally, they had to report a minimum of 100 lifetime occasions of either crystalline MDMA or ecstasy pill use. For the data collection in 2019-2021, the inclusion criteria were defined by each participant having consumed MDMA a minimum of 25 times in life, with the additional stipulation of having consumed MDMA at least once within the preceding four months. These less restrictive inclusion criteria were applied because of the COVID-19 pandemic taking place during that timeframe, which led to reduced MDMA consumption rates.[1] Smoking status was assigned to participants who had smoked over 100 cigarettes lifetime and smoked within the past six months.

Ultimately, the final analysis included 36 subjects from the investigation in 2015-2016 (17 users, 19 CON) and 86 subjects from the 2019-2021 study (44 users, 42 CON). In the final dataset, the only missing data pertained to depression scores, with 10 MDMA users and 12 CON subjects lacking this information.

Statistical analyses

Demographics and substance use

To evaluate differences in demographic variables and substance use between groups, Wilcoxon or Chi-Square tests were applied to the variables sex, age, years of education, ADHD score, verbal IQ, and depression score.

Group differences in structural imaging

The models pertaining to the globus pallidus, amygdala, pars triangularis, rostral anterior cingulate, cuneus, fimbra, and insula exhibited violations of normality assumptions. Therefore, permutation testing was applied for these regions.

Group differences in memory performance

For each memory model, normality and homoscedasticity assumptions were tested using the Shapiro-Wilk and the Breusch-Pagan test, respectively. For permutation tests, the "Exact"

method, which generates all permutations of the dependent variable, was used along with the unique sum of squares. For all ANCOVA models, type 3 sums of squares were calculated. Given that sex differences have been proposed regarding the susceptibility to MDMA-related cognitive toxicity[2,3], the sex*group interactions were also tested in the models. However, they never became significant and were therefore dropped from the final models.

Correlation with PET-derived 5-HT receptor and transporter maps

Regarding the associations of cortical and subcortical grey matter differences and 5-HT receptor distributions, normality distribution was tested, and Pearson or Spearman correlations were applied accordingly. The correlations for the cortical and the subcortical regions were carried out separately in case 5-HT receptor distributions between cortical and subcortical regions were shown to vary substantially[4], which was the case for all except the 5-HT1A receptors. The correlation of values across cortical and subcortical regions may present methodological issues, such as bimodality, if the data points are distributed around two distinct values. To address the possible impact of spatial autocorrelation, we statistically verified the results by applying spin tests using the Alexander Bloch method.[5] This method accounts for spatial dependencies in neuroimaging data by rotating the data in space to generate randomized versions, ensuring that the observed effects are not due to nearby regions' inherent correlations. Calculations were carried out with neuromaps (version 0.0.5+27.ga89b699).[6]

Supplementary Table 1: Brain regional group differences

**Brain regions**	***Estimate***	***SE***	***95% CI***		***p***
			LL	***UL***	
**Subcortical regions**					
Amygdala	-0.012	0.015	-0.042	0.018	.422
Globus Pallidus	-0.014	0.007	-0.028	0.000	.053
Hippocampus CA1	-0.036	0.009	-0.053	-0.018	**<.001***
Hippocampus CA2/3	-0.006	0.002	-0.010	-0.002	**.002***
Hippocampus CA4	-0.015	0.007	-0.029	-0.002	**.025***
Mammillary body	0.001	0.001	-0.001	0.002	.483
Stratum	-0.007	0.005	-0.017	0.004	.199
Subiculum	0.003	0.003	-0.004	0.009	.403
**Cortical regions**					
Banks of the superior temporal sulcus	-0.027	0.021	-0.070	0.015	.202
Caudal anterior cingulate cortex	0.007	0.027	-0.047	0.061	.794
Caudal middle frontal gyrus	-0.004	0.017	-0.038	0.030	.810
Cuneus	-0.007	0.016	-0.040	0.025	.650
Entorhinal cortex	0.055	0.036	-0.017	0.126	.131
Frontal pole	-0.013	0.023	-0.058	0.032	.563
Fusiform gyrus	-0.005	0.017	-0.039	0.029	.755

Inferior parietal cortex	-0.018	0.016	-0.050	0.014	.257
Inferior temporal gyrus	0.000	0.016	-0.031	0.031	.999
Insula	-0.041	0.033	-0.106	0.024	.214
Isthmus cingulate cortex	-0.058	0.022	-0.102	0.014	**.010***
Lateral occipital cortex	-0.028	0.012	-0.053	-0.004	**.023***
Lateral orbitofrontal cortex	0.002	0.016	-0.030	0.033	.909
Lingual gyrus	-0.005	0.015	-0.035	0.025	.732
Medial orbitofrontal cortex	-0.019	0.015	-0.049	0.011	.209
Middle temporal gyrus	0.009	0.019	-0.029	0.046	.647
Parahippocampal gyrus	-0.028	0.026	-0.081	0.024	.280
Paracentral gyrus	-0.015	0.018	-0.050	0.021	.411
Pars opercularis	-0.016	0.017	-0.050	0.017	.339
Pars orbitalis	-0.013	0.021	-0.055	0.029	.542
Pars triangularis	-0.022	0.016	-0.054	0.009	.167
Pericalcarine cortex	-0.035	0.020	-0.074	0.004	.075
Postcentral gyrus	-0.012	0.016	-0.043	0.019	.455
Posterior cingulate cortex	-0.021	0.018	-0.056	0.015	.254
Precentral gyrus	0.004	0.017	-0.029	0.037	.792
Precuneus	-0.019	0.016	-0.050	0.013	.237
Rostral anterior cingulate cortex	0.023	0.021	-0.019	0.065	.286
Rostral middle frontal cortex	-0.014	0.015	-0.043	0.015	.351
Superior frontal gyrus	-0.010	0.017	-0.045	0.024	.553
Superior parietal gyrus	0.004	0.015	-0.025	0.033	.779
Superior temporal gyrus	-0.026	0.018	-0.061	0.009	.145
Supramarginal gyrus	-0.019	0.015	-0.049	0.011	.204
Temporal pole	-0.008	0.036	-0.080	0.064	.825
Transverse temporal gyrus	0.004	0.029	-0.054	0.063	.883

Results of linear regression analyses for all 42 brain regions, showing group effects. The user group serves as the reference category, and the estimates represent the difference compared to the control group. For subcortical regions, total intracranial volume (TIV) was included as an additional covariate.
P-values that are significant at the uncorrected level are shown in bold and marked with an asterisk (*).

Supplementary Table 2: Detailed regression results of significant regions

**Brain regions**	***Estimate***	***SE***	***95% CI***		***p***	***p*-corrected**	***f²***
			LL	***UL***			
**Hippocampus CA1**							
Group	-0.036	0.009	-0.053	-0.018	<.000***	**.027***	0.14
TIV	0.000	0.000	0.000	0.000	<.000***		
Age	0.000	0.001	-0.002	0.001	.492		
Sex	-0.022	0.012	-0.045	0.001	.064		
Alcohol	0.000	0.003	-0.005	0.005	.964		
Cannabis	0.016	0.018	-0.019	0.052	.355		
Nicotine	0.004	0.004	-0.004	0.012	.291		
**Hippocampus CA2/3**							
Group	-0.006	0.002	-0.010	-0.002	.002**	**.042***	0.09
TIV	0.000	0.000	0.000	0.000	<.000***		
Age	0.000	0.000	0.000	0.000	.871		
Sex	0.000	0.002	-0.005	0.005	.985		
Alcohol	0.001	0.001	0.000	0.002	.252		
Cannabis	0.006	0.004	-0.002	0.013	.143		
Nicotine	0.001	0.001	-0.001	0.002	.385		
**Hippocampus CA4**							

Group	-0.015	0.007	-0.029	-0.002	<.025*	.213	0.04
TIV	0.000	0.000	0.000	0.000	<.000***		
Age	0.000	0.000	-0.001	0.000	.351		
Sex	-0.013	0.009	-0.031	0.004	.137		
Alcohol	-0.001	0.002	-0.004	0.003	.786		
Cannabis	0.006	0.013	-0.021	0.033	.657		
Nicotine	0.005	0.003	-0.001	0.011	.085		
**Isthmus cingulate gyrus**							
Group	-0.058	0.022	-0.102	-0.014	.009**	.134	0.06
Age	-0.006	0.002	-0.009	-0.003	<.000***		
Sex	-0.056	0.02	-0.097	-0.016	.007**		
Alcohol	0.004	0.006	-0.008	0.016	.525		
Cannabis	-0.064	0.044	-0.15	0.022	.145		
Nicotine	0.001	0.010	-0.018	0.021	.892		
**Lateral occipital cortex**							
Group	-0.028	0.012	-0.053	-0.004	.023*	.213	0.04
Age	-0.003	0.001	-0.005	-0.001	<.000***		
Sex	-0.002	0.011	-0.025	0.021	.858		
Alcohol	0.004	0.003	-0.002	0.011	.197		
Cannabis	0.015	0.024	-0.034	0.063	.549		
Nicotine	-0.006	0.006	-0.017	0.005	.252		

Detailed linear regression results and effect sizes (partial eta^2) of regions exhibiting significance at the uncorrected level. The hippocampal CA1 and CA2/3 regions remained significant after applying Benjamini-Hochberg-correction for 42 comparisons or models, respectively.

Supplementary Table 3: Memory differences between groups

ANCOVA Results

**Memory Score**	***Sum Sq***	***df***	***F*-value**	***P***	***p*-corrected**	***f*^{2}**
**Supraspan**						
Group	9.51	1	2.15	.145	.332	0.02
Sex	1.31	1	0.30	.587		0.00
Verbal IQ	60.08	1	13.59	**<.001**		0.12
Age	18.15	1	4.10	**.045**		0.04
Cannabis	0.05	1	0.01	.913		0.00
Alcohol	3.31	1	0.75	.389		0.01
Nicotine	19.60	1	4.43	**.038**		0.04

PERM ANCOVA Results

**Memory Score**	***Sum Sq***	***df***	***F*-value**	***P***	***p*-corrected**	***f*^{2}**
**Learning performance**						
Group	288	1	6.25	**.014**	**.040**	0.06
Sex	53	1	1.16	.284		0.01
Verbal IQ	861	1	18.69	**<.001**		0.18
Age	54	1	1.17	.282		0.01
Cannabis	10	1	0.21	.647		0.00
Alcohol	0	1	0.00	.998		0.00
Nicotine	300	1	6.51	**.012**		0.06
**Recall after interference**						
Group	65.35	1	16.450	**<.001**	**.001**	0.16
Sex	18.00	1	4.529	**.036**		0.04
Verbal IQ	53.91	1	13.562	**<.001**		0.12
Age	3.42	1	0.861	.355		0.01
Cannabis	2.34	1	0.589	.444		0.01
Alcohol	1.24	1	0.311	.578		0.00

Nicotine	7.02	1	1.764	.187		0.02
**Recall after 2h**						
Group	58.08	1	12.54	**<.001**	**.002**	0.12
Sex	1.04	1	0.23	.636		0.10
Verbal IQ	49.09	1	10.60	**.002**		0.09
Age	0.07	1	0.01	.906		0.00
Cannabis	10.27	1	2.22	.139		0.02
Alcohol	0.45	1	0.10	.755		0.00
Nicotine	8.73	1	1.89	.173		0.02
**Recognition**						
Group	0.17	1	21.83	**<.001**	**<.001**	0.20
Sex	0.02	1	2.22	.139		0.02
Verbal IQ	0.10	1	12.23	.001		0.11
Age	0.01	1	2.13	.148		0.02
Cannabis	0.01	1	0.69	.408		0.01
Alcohol	0.00	1	0.00	.955		0.00
Nicotine	0.07	1	8.06	**.005**		0.08

For supraspan an ANCOVA model with type III SS was applied. For learning performance, recall after interference, and recall after 2h, and recognition, permutation testing with type III SS were calculated, because the normality of residuals assumption was not met. *P*-values for group effects were Benjamini-Hochberg-corrected for 5 different comparisons or models, respectively.

Supplementary Table 4: Association CA1 VBM with Memory Scores Results

ANCOVA Results

**Memory Score**	***Sum Sq***	***df***	***F*-value**	***P***	***p*-corrected**	***f*²**
**Supraspan**						
CA1 VBM	0.62	1	0.14	.0.71	.711	0.00
Sex	0.03	1	0.01	.936		0.00
Verbal IQ	57.43	1	12.75	**.001**		0.12
Age	21.09	1	4.68	**.033**		0.04
Cannabis	0.76	1	0.17	.682		0.00
Alcohol	7.01	1	1.56	.216		0.01
Nicotine	21.03	1	4.76	**.031**		0.03

PERM ANCOVA Results

**Memory Score**	***Sum Sq***	***df***	***F*-value**	***P***	***p*-corrected**	***f*²**
**Learning Performance**						
CA1 VBM	27	1	0.54	.461	.576	0.00
Sex	50	1	1.03	.313		0.00
Verbal IQ	788	1	16.03	**<.001**		0.15
Age	66	1	1.37	.245		0.01
Cannabis	46	1	0.94	.333		0.00
Alcohol	16	1	0.33	.569		0.00
Nicotine	226	1	4.65	**.033**		0.04
**Recall Interference**						
CA1 VBM	12.17	1	5.53	**.021**	.051	0.05
Sex	28.85	1	6.6	**.012**		0.06
Verbal IQ	44.21	1	10.12	**.001**		0.10
Age	2.95	1	0.67	.423		0.00
Cannabis	11.14	1	2.55	.113		0.02
Alcohol	0.40	1	0.09	.761		0.00
Nicotine	2.04	1	0.47	.496		0.00
**Recall 2h**						
CA1 VBM	27.1	1	5.51	**.021**	.051	0.05

Sex	8.1	1	1.64	.203		0.02
Verbal IQ	39.1	1	7.94	**.006**		0.08
Age	0.0	1	0.01	.921		0.00
Cannabis	23.4	1	4.75	**.031**		0.04
Alcohol	0.9	1	0.19	.662		0.00
Nicotine	3.2	1	0.64	.424		0.00
**Recognition**						
CA1 VBM	0.01	1	1.11	.293	.489	0.01
Sex	0.01	1	1.44	.233		0.01
Verbal IQ	0.08	1	8.36	**.005**		0.08
Age	0.02	1	2.32	.131		0.02
Cannabis	0.03	1	2.66	.106		0.02
Alcohol	0.01	1	0.94	.334		0.00
Nicotine	0.04	1	4.05	**.047**		0.04

For Supraspan an ANCOVA model with Type III SS was applied. For Learning Performance, Recall after Interference, and Recall after 2h, Permutation Testing with Type III SS was conducted, since the normality of residuals assumption was not met.

Supplementary Table 5: Association CA2/3 VBM with Memory Scores Results

ANCOVA Results

**Memory Score**	***Sum Sq***	***df***	***F*-value**	***P***	***p*-corrected**	f^2
**Supraspan**						
CA2/3 VBM	2.07	1	0.46	.498	.498	0.00
Sex	1.55	1	0.34	.558		0.00
Verbal IQ	56.8	1	12.65	**.001**		0.12
Age	19.7	1	4.38	**.039**		0.04
Cannabis	0.96	1	0.21	.645		0.00
Alcohol	7.64	1	1.70	.194		0.02
Nicotine	16.26	1	3.62	.059		0.03

PERM ANCOVA Results

**Memory Score**	***Sum Sq***	***df***	***F*-value**	***P***	***p*-corrected**	f^2
**Learning Performance**						
CA2/3 VBM	149	1	3.16	.079	.123	0.03
Sex	90	1	1.90	.171		0.02
Verbal IQ	790	1	16.68	**.000**		0.15
Age	64	1	1.36	.247		0.01
Cannabis	57	1	1.21	.274		0.01
Alcohol	29	1	0.61	.435		0.00
Nicotine	227	1	4.79	**.031**		0.04
**Recall Interference**						
CA2/3 VBM	22.24	1	5.07	**.026**	.066	0.06
Sex	23	1	5.24	**.024**		0.06
Verbal IQ	47.47	1	10.82	**.001**		0.10
Age	2.12	1	0.48	.489		0.00
Cannabis	13.05	1	2.97	.088		0.02
Alcohol	1.62	1	0.37	.545		0.04
Nicotine	2.25	1	0.51	.475		0.03
**Recall 2h**						
CA2/3 VBM	35.1	1	7.24	**.008**	**.041**	0.06
Sex	5.2	1	1.08	.301		0.01
Verbal IQ	42.2	1	8.70	**.004**		0.09
Age	0.2	1	0.04	.844		0.00
Cannabis	26.9	1	5.54	**.020**		0.05

Alcohol	3.0	1	0.62	.433		0.00
Nicotine	3.4	1	0.70	.406		0.00
**Recognition**						
CA2/3 VBM	0.03	1	2.79	.098	.123	0.03
Sex	0.02	1	1.82	.180		0.02
Verbal IQ	0.08	1	8.77	**.004**		0.09
Age	0.02	1	2.42	.123		0.02
Cannabis	0.01	1	3.05	.084		0.03
Alcohol	0.01	1	1.37	.244		0.01
Nicotine	0.04	1	4.13	.045		0.04

For Supraspan an ANCOVA model with Type III SS was applied. For Learning Performance, Recall after Interference, and Recall after 2h, Permutation Testing with Type III SS was conducted, since the normality of residuals assumption was not met.

Supplementary Table 6: Correlation Results

	Method	*Estimate*	*p*	*95% CI*
**Cortical Regions**				
SERT	Spearman	0.218	.216	[-0.130, 0.518]
5-HT$_{1A}$	Pearson	-0.404	**.017**	[-0.653, -0.076]
5-HT$_{1B}$	Spearman	0.098	.582	[-0.249, 0.422]
5-HT$_{2A}$	Spearman	-0.234	.182	[-0.531, 0.113]
5-HT$_{4}$	Pearson	-0.350	**.042**	[-0.615, -0.013]
**Subcortical Regions**				
SERT	Pearson	0.111	.732	[-0.495, 0.644]
5-HT$_{1A}$	Pearson	-0.073	.822	[-0.621, 0.523]
5-HT$_{1B}$	Spearman	0.413	.185	[0.086, 0.659]
5-HT$_{2A}$	Spearman	0.643	**.028**	[0.390, 0.806]
5-HT$_{4}$	Spearman	0.278	.379	[-0.065, 0.564]

Details of correlation of cortical and subcortical grey matter differences with receptor distributions results. For Spearman correlations, Fisher Z-transformed confidence intervals are reported.

Supplementary Table 7: Spatial autocorrelation corrected results

Cortical Regions	Method	*Estimate*	*p*
SERT	Pearson	-0.121	.494
5-HT$_{1A}$	Pearson	-0.401	**.017**
5-HT$_{1B}$	Pearson	0.156	.360
5-HT$_{2A}$	Pearson	-0.028	.864
5-HT$_{4}$	Pearson	-0.379	**.018**

Spin method results to address spatial autocorrelation for cortical regions.

Supplementary Literature

1. Mellos E, Paparrigopoulos T. Substance use during the COVID-19 pandemic: What is really happening? *Psychiatrike*. 2022;33(1):17-20. doi:10.22365/jpsych.2022.072

2. Von Geusau NA, Stalenhoef P, Huizinga M, Snel J, Ridderinkhof KR. Impaired executive function in male MDMA ("ecstacy") users. *Psychopharmacology (Berl)*. 2004;175(3):331-341. doi:10.1007/S00213-004-1832-8

3. Price JS, Shear P, Lisdahl KM. Ecstasy Exposure & Gender: Examining Components of Verbal Memory Functioning. *PLoS One*. 2014;9(12):e115645. doi:10.1371/JOURNAL.PONE.0115645

4. Varnäs K, Halldin C, Hall H. Autoradiographic distribution of serotonin transporters and receptor subtypes in human brain. *Hum Brain Mapp*. 2004;22(3):246-260. doi:10.1002/HBM.20035

5. Alexander-Bloch AF, Shou H, Liu S, et al. On testing for spatial correspondence between maps of human brain structure and function. *Neuroimage*. 2018;178:540-551. doi:10.1016/J.NEUROIMAGE.2018.05.070

6. Markello RD, Hansen JY, Liu ZQ, et al. neuromaps: structural and functional interpretation of brain maps. *Nat Methods 2022 1911*. 2022;19(11):1472-1479. doi:10.1038/s41592-022-01625-w
